# Watchful waiting or induction of labour – a matter of informed choice: identification, analysis and critical appraisal of decision aids and patient information regarding care options for women with uncomplicated singleton late and post term pregnancies: a review

**DOI:** 10.1186/s12906-015-0663-y

**Published:** 2015-05-07

**Authors:** Bettina Berger, Christiane Schwarz, Peter Heusser

**Affiliations:** Institute of Integrative Medicine, Chair for Theory of Medicine, Integrative and Anthroposophic Medicine, Witten/Herdecke University, Gerhard-Kienle-Weg 4, 58313 Herdecke, Germany; Hanover Medical School, Midwifery Research Unit, Hannover, Germany

**Keywords:** Patient preferences, Complementary and alternative medicine, CAM, Late term pregnancy, Post term pregnancy, Decision aid, Patient information, Evidence-based, Informed choice, Systematic review, Development and evaluation of complex intervention

## Abstract

**Background:**

Decision-making during pregnancy regarding different options of care can be difficult, particularly when risks of intervention versus no intervention for mother and baby are unclear. Unbiased information and support for decision making may be beneficial in these situations. The management of normal pregnancies at and beyond term is an example of such a situation. In order to determine the need to develop an evidence-based decision aid this paper searches, analyses and appraises patient decision aids and patient information leaflets regarding care options in cases of late term and post-term pregnancies, including complementary and alternative medicine (CAM).

**Methods:**

A literature search was carried out in a variety of lay and medical databases. Inclusion criteria: written information related to uncomplicated singleton pregnancies and targeted at lay people. Analysis and appraisal of included material by means of quality criteria was set up based on the International Patient Decision Aid Standards accounting for evidence-basing of CAM options.

**Results:**

Inclusion of two decision aids and eleven leaflets from four decision aids and sixteen leaflets. One decision aid met the quality criteria almost completely, the other one only insufficiently despite providing some helpful information. Only one leaflet is of good quality, but cannot substitute a decision aid.

**Conclusions:**

There is an urgent need for the design of an evidence-based decision aid of good quality for late-term or post-term pregnancy, particularly in German language.

**Electronic supplementary material:**

The online version of this article (doi:10.1186/s12906-015-0663-y) contains supplementary material, which is available to authorized users.

## Background

Involvement of patients and consideration of their preferences in the treatment decision has been demanded for care options with unclear outcomes. However, the management of pregnancy at and beyond term has been a topic of debate worldwide within the medical and midwifery community for many decades. The controversial discussion concerns the pros and cons of induction of labour in the light of the medicalization of the “natural” birth, the difficulties to identify the appropriate length of human gestation and the definition whether or not a pregnancy is “overdue”, “post-date” or “prolonged”[1-4]. The available data have been discussed as insufficient to demonstrate that routine induction of labour is superior to an expectant management to reduce maternal and perinatal mortality and morbidity [3,5]. The meta-analysis of Wennerholm illustrated statistical problems with rare outcomes such as perinatal mortality. The optimal management of pregnancies at 41 weeks and beyond is thus unknown [6]. Therefore, the involvement of women and their preferences in the treatment decision has been demanded [7-9].

Perinatal mortality in developed countries is low. Nevertheless, there is an ongoing debate on whether some perinatal deaths, particularly stillbirths, may be avoidable [10-12]. In the absence of solid knowledge on causes and prevention of fetal death, one strategy that has internationally been adopted is to terminate pregnancy at or beyond term, as fetal death rates seem to rise once the estimated date of delivery has been reached. In recent years various medical associations have issued guidance on when and how to recommend induction of labour (IOL)[13-15]. But a recent overview of the quality and recommendations of guidelines for the management of term and post-term pregnancy shows, that recommendations for induction of labour differ [16]. Internationally, there is no consensus about when and how to routinely observe fetal wellbeing once term is reached. This may explain some of the uncertainties regarding different options of care [3,4].

In 2010 the German Society of Gynecology and Obstetrics (Deutsche Gesellschaft für Gynäkologie und Geburtshilfe, DGGG) issued a consensus guideline regarding options of care for women with uncomplicated singleton pregnancies continuing beyond the estimated due date (EDD) [15]. However, recommendations mainly refer to randomized controlled trials from 1969 to the early 21^st^ century which have been analyzed in a meta-analysis by Gülmezoglu et al. [11] suggesting IOL at 41 completed weeks may significantly reduce perinatal mortality and the risk of neonatal morbidity without increasing the caesarean section rate. From these findings, the guideline concludes that there are substantial reasons to generally offer IOL after 41 completed weeks of pregnancy, and to strongly recommend IOL after 41 + 3 weeks. The new recommendation refers to a recently published retrospective analysis of German routine perinatal data, indicating that the risk for fetal death in pregnancies going beyond term is remarkably lower than found in studies published about the situation from other countries [3]. These results do not support the recommendation of routine induction of labor in low risk pregnancies at 41+ 0/7 weeks of pregnancy.

Relevant literature shows a diversity of opinions on how and to which extent pregnant women should be informed of conflicting evidence on treatment options they will have to decide on. Recommendations regarding patient information postulate that uncertainties need to be addressed to patients [17]. But there is a possibility that this imposes a dilemma on them, as there is only the choice between risks that cannot be quantified [18]. Decision aids are defined as tools to help people participating in their health decisions in ways they prefer. They aim to provide facts about a person’s condition, the options and their features, help people to clarify their values and help them to share their values with their health care practitioner and others, in order for a course of action to be planned that matches their values. Patient decision aids do not advise people to choose one option over another. They do not replace counseling from a health care practitioner. Instead, they prepare people to discuss the options with their health care practitioner [19].

### Informational and decision support needs of pregnant women

There is some evidence that women during pregnancy are not always informed or included in decision-making. Thompson conducted a survey in Queensland, Australia, about the perceived information about risks and benefits of procedures and the role of decision making about the procedures. 3,542 eligible women (34.2%) completed the survey. Between 4% and 60% of women reported that they had not been informed of the benefits and risks of the procedure they experienced. Between 2% and 34% of women reported not having been consulted in decision-making. Over one quarter (26%) of the women who had an episiotomy reported neither having been informed nor consulted prior to the procedure. Thomson concluded that there is an urgent need for interventions that facilitate information provision and consumer involvement in decision-making about several perinatal procedures, especially those performed within the time-limited intrapartum care episode [20]. Decisions concerning the management of post-term pregnancy have been studied only in a few studies. Dependent on the situation or the cultural context, attitudes and preferences regarding the main options of post-term women differ. The clinical management options for healthy pregnant women at and beyond term are watchful waiting, or induction of labour [12]. Watchful waiting describes a range of regimen for monitoring fetal wellbeing, including electronic fetal monitoring (CTG), ultrasound assessment of amniotic fluid volume, and monitoring of fetal movements at different times past 40 completed gestational weeks. For induction of labour, there are several methods available. Besides medical means like cervical ripening and stimulation of contractions with prostaglandines or oxytocin [21], labour may be provoked by mechanical (amniotomy, membrane sweep) [22] or complementary (raspberry leaves, castor oil, acupuncture) methods [23-28].

Stevens developed a trial comparing two different counselling styles to explore how to best include pregnant women in the decision regarding post-term management. She found that women were better able to realize informed decision-making when they were supported by non-directive compared to directive counselling [29]. Westfall conducted a qualitative study with childbearing women about their perspectives on prolonged pregnancy and induction of labour. Participants of this study initially had objections against induction of labour; however, once they had passed the “estimated date of delivery”, they changed their minds and used complementary self-help measures to start labour. This does not so much reflect concerns regarding fetal wellbeing, but the inconvenience of being pregnant [30]. Roberts had the intention to invite women to consider conservative treatment, but most women were unwilling to accept the conservative management [9]. Gatward and colleagues were interested in the fears and worries of the included women. Participants of the natural birth group were mainly worried about the potential impact of induction on the baby, whereas the induction group expressed concern about the effect on themselves and deprivation of a natural birth. Worries in both groups were dissipated by successfully birthing a healthy baby. But also a lack of meaningful information was found [8]. In a trial on how information and role in decision-making influence women’s preferences in cases of prolonged pregnancies, Stevens et al. came to the conclusion that the findings highlight the potential value of strategies such as patient decision aids and health care professional education to improve the quality of information available to women and their capacity for informed decision-making during pregnancy and birth [29]. Heimstad conducted a trial where women in the 41st gestational week were randomized to a waiting group or an induction group and asked about their attitudes towards post-term pregnancy [7]. No relevant differences in the outcomes related to morbidity of infants or mothers could be found. In the induction group, only 74% of women said they would prefer the same management in future pregnancies; also 38% of women who had serial antenatal monitoring would prefer this option again. Therefore it was stated, that in case of uncomplicated pregnancy and continued surveillance, women’s own wishes may guide the decision to induce or monitor a pregnancy beyond 41 weeks. However, for this decision the adequate information materials have to be available. Patient decision aids, as special types of patient information, are particularly appropriate to facilitate patients’ involvement in decision- making and to help patients to deliberate the advantages and disadvantages of different options of care [31]. Accordingly, we judge a patient decision aid to be a possible tool, supporting pregnant women and health care professionals for the involvement of pregnant women in the obstetric management of pregnancies continuing beyond the EDD and for their participation in informed decision-making with regard to the various options of care.

### Information about and the use of complementary and alternative medicine (CAM)

During pregnancy, depending on the definition of CAM, between 1% and 87% of pregnant women use complementary and alternative medicine according to a very recent literature review by Hall et al. [32]. In United States, a survey among pregnant women with a response rate of 74.3% showed, that in 2013 68.5% of participants reported CAM use during their pregnancies. The authors concluded, that by this given frequency of CAM use obstetrical providers should be informed about CAM and incorporate discussions about its use into routine patient assessments [33]. Complementary and alternative medicine are also increasingly popular amongst midwives in different countries [34]. Also the inquiry of Steel in Australia among 1 800 pregnant women emphasized the necessity for a collaborative approach of interactions between pregnant women, conventional maternity health providers and CAM practitioners to accommodate appropriate information transferal and coordinated maternity care [35].

Reasons why people use complementary medicine have been studied widely. One main reason is the wish to find methods and ways to become active as a patient, and be able to influence one’s own health [36]. This intention should be taken into account when regarding the inconclusive evidence concerning CAM methods to support induction of labour. Only a small number of studies have been conducted worldwide, and few systematic reviews have been published. Kavanagh et al. only found one small study on the effect of sexual intercourse on labour for their 2008 Cochrane review [23]. The result is inconclusive; the small study was underpowered. As far as breast stimulation for the same purpose is concerned, the same research team found six trials for inclusion in their 2009 Cochrane review. Breast stimulation appears beneficial for reducing the number of women not in labour after 72 hours and seems to have a protective effect on postpartum hemorrhage [25]. Smith et al. updated a Cochrane review on homeopathy for inducing labour. They found that the demand for homeopathy among pregnant women is high, but the body of evidence is poor [27]. Hall examined the evidence on multiple methods to induce labour and stated that in spite of women demanding CAM there is a lack of research. However, acupuncture, raspberry leaf tea and breast stimulation appear beneficial [28]. For castor oil, three studies were included in a 2013 Cochrane review. All were of poor methodological quality and did not produce any significant results [24]. Another 2013 Cochrane review on acupuncture for IOL reported inconsistent results of the 14 RCTs that they included [26]. All authors agreed on the urgent need for more well designed studies on the topic, as pregnant women have a particular interest in CAM.

In 2014 Steel et al. conducted a nationwide survey among women and their CAM use. The participants’ responses were analyzed to examine the relationship between the use of CAM and adverse birth outcomes from their most recent pregnancy. Of the respondents (n = 1835; 79.2%), there were variations in birth outcomes for the women who used different forms of CAM. Notably, the outcome which was most commonly associated with CAM use was emotional distress. This was found to occur more commonly in women who practiced meditation/yoga at home, used flower essences, or consulted with a chiropractor. In contrast, women who consulted with a chiropractor or consumed herbal teas were less likely to report a premature birth, whilst participation in yoga classes was associated with an increased incidence of post-partum/intra partum haemorrhage [35]. Even when women use complementary medicine they want to receive evidence-based patient information about risks and missing evidence in the case of CAM options [37]. Until now, CAM options have rarely been included into the information and counseling tools like evidence-based decision aids.

We conclude that information on CAM for induction of labour needs to be part of a person- centred, evidence-based decision aid. Therefore, this article identifies, analyzes and appraises existing decision aids regarding the obstetric management of pregnancies continuing beyond the estimated day of delivery (EDD), including CAM-options, in order to assess the need for the development of an additional decision aid.

## Methods

### Literature search

We took an inventory of existing decision aids for the obstetric management of late term (40 + 1 to 41 + 6 weeks of gestation) and post term (≥42 + 0 weeks of gestation) pregnancies. Inclusion criteria: a) decision aids or other printed information material for pregnant women, b) published in German or English language, c) available on the Internet.

Exclusion criteria: a) Information for other groups, for example midwives, b) in languages other than German or English, c) informally designed patient information or decision aids. The identified decision aids were analyzed and appraised by means of quality criteria, which we set up on the basis of the “IPDAS patient decision aid user checklist” [38].

Since it is known that decision aids do not always use evidence-based material [18], we decided also to include other relevant types of patient information into our examination such as information sheets, leaflets and patient handouts - which cannot be classified as decision aids in the proper sense. Our aim was to be able to include evidence-based patient information material, which can offer some relevant aspects for the development of an evidence-based decision aid.

In order to compile an inventory of relevant English- and German-language decision aids and other types of patient information, we conducted a systematic literature search at the beginning of 2012. Results were updated in early 2013. We also searched for existing evaluations of available information, yet without any findings.

The following databases, search engines and websites were searched, among them deliberately also databases predominantly targeted at lay people and thus particularly accessible for pregnant women as well: AAFP (American Academy of Family Physicians), AWMF (Arbeitsgemeinschaft der Wissenschaftlichen Medizinischen Fachgesellschaften e. V.), BZgA (Bundeszentrale für gesundheitliche Aufklärung), Cochrane Library, DIMDI (Deutsches Institut für Medizinische Dokumentation und Information), DHV (Deutscher HebammenVerband e. V.), GOOGLE, GOOGLE scholar, Gesundheitsinformationen des IQWIG (Institut für Qualität und Wirtschaftlichkeit im Gesundheitswesen), MEDLINE incl. Deutsches Ärzteblatt, MEDLINE PLUS, MEDPILOT, MIDIRS (Midwives Information and Resource Service), NHS (National Health Service) incl. NICE (National Institute for Health and Clinical Excellence), OHRI (Ottawa Hospital Research Institute), PubMed (incl. MeSH- Terms), PubMed Health, UpToDate, WHO/HEN.

The following search terms were used:

- English search terms and combinations:

(“post-term” OR “postterm” OR “overdue” OR “induction of labor” OR “after due date” OR “labor, induced” [MeSH] OR “delay of birth” OR “target date” OR “delivery date” OR “watchful waiting”[MeSH] OR “due day” OR “extended duration of pregnancy” OR “postmaturity” OR “prolonged pregnancy” OR “expectant management” OR “Pregnancy, Prolonged”[MeSH] OR “term birth”[MeSH] OR

“estimated due date” OR “EDD” OR “beyond EDD” OR “IOL”) AND (“patient handout” OR “patient hand out” OR “patient decision aid” OR “patient aid” OR “patient information” OR “PDA” OR “decision guidance” OR “decision support” OR “decision-making aid” OR “decision-making help” OR “decision-making support” OR

“patient education handout”[MeSH] OR “decision aid” OR “informed choice” OR

“guidance for patient”)

- German search terms and combinations:

(“Terminüberschreitung” OR “Übertragung” OR “Einleitung der Geburt” OR

“Geburtseinleitung” OR “Geburtstermin” OR “Errechneter Termin” OR

“Entbindungstermin”) AND (“Entscheidungshilfe” OR “Patienteninformation” OR

“Informierte Entscheidung” OR “Patientenleitlinie” OR “Patientenhilfe”)

#### Inclusion criteria for assessment

We included decision aids and other types of information for pregnant women into our assessment which refer to uncomplicated singleton pregnancies continuing beyond the EDD and which are addressed to medical lay people, i.e. primarily to pregnant women themselves. Of the various types of information for pregnant women only those which comply with the IPDAS definition cited above were classified as decision aids.

#### Exclusion criteria for assessment

We excluded decision aids and other types of information for pregnant women which refer to IOL, but which explicitly do not cover the indications of late and post term pregnancies. We also excluded information on IOL whose reference to late and post term pregnancies is confined to merely listing those terms as possible indications for IOL without giving any further information.

The search, the application of the inclusion and exclusion criteria and the classification of patient information as decision aids or other types of patient information were carried out independently by two authors (CS, BB). Discrepancies were resolved by discussion.

### Critical appraisal

For the analysis of the identified decision aids and other types of patient information, we designed a checklist. To this end the design of our checklist was based on the criteria relating to content and development process set out in the “IPDAS patient decision aid user checklist” [38]. The checklist has been developed in cooperation with gynecologists and midwiferies.

We included all of the I. Section (Content Criteria) without the criteria related to tests. We then tailored the checklist for the specific demands of the decisional situation of pregnant women at and beyond term (No.1.2.1-1.2.3;1.2.5, see Additional file 1). We added two further questions (No. 4.4; No. 4.5) to ask for further references and further support services, because of the evidence for further communicative support needs [37]. In consideration of the interest among many pregnant women in complementary and alternative approaches, the supply of evidence-based information on the effectiveness or ineffectiveness of care options from the field of complementary and alternative medicine (CAM) was included as a further quality criterion (No.1.6, see Additional file 1). We looked at those treatment options that are commonly used in maternity care context and evaluated those in relation to existing or missing evidence [17]. The following criteria have been added: Does it refer to natural and complementary or alternative (CAM) methods of labour stimulation? (No. 1.6.1. membrane sweep/No 1.6.2. sexual intercourse/ No 1.6.3. nipple stimulation, No 1.6.4. CAM methods (e. g. castor oil, raspberry leaf tea, acupuncture)).

We added question No. 1.9 about references to relevant guideline recommendations to relate the decision aid to the health care system of the users, because patients might be afraid of discussing evidence with their health care providers and should be informed about medical guidelines as well.

In the section II. (Developmental process) we added question No. 2.1 about naming the developer and deleted the question about reporting steps to find, appraise, summarize evidence, because further educational intervention would have been necessary to develop this abilities in lay persons [19]. The language level was judged according to the sub-criteria listed under “Does the decision aid use plain language?” on p. 8 of Additional file 1. The checklist was discussed with several obstetricians and with specialists in evidence-based patient information. Section III (Effectiveness) we could not apply, because we could not identify any evaluation studies related to the identified decision aids. Our checklist contains 49 criteria, of which 30 refer to content and 19 on the development process of the decision-aids and information. Some of the criteria were broken down further into sub criteria (Additional file 1). The checklist was then used to analyze and appraise the decision-aids as well as the information for pregnant women. The information leaflets for pregnant women describe the situation and options of care (IOL versus watchful waiting) for pregnant women at and beyond term, but do not particularly facilitate a decisional process. This is why some of our appraisal criteria may not strictly be applicable. Nonetheless, we decided to also apply the checklist to types of information other than decision aids in order to assess to which extent those would also be capable of facilitating pregnant women’s decision-making process. The authors (CS, BB) independently analyzed and appraised the decision aids and other kinds of information for pregnant women. Discrepancies were resolved by discussion.

### Ethical approval

No experimental research has been done and no patients have been involved. Only patient information material was analyzed. Therefore no ethical approval was obtained and no informed consent procedure was realized.

## Results

### Main findings

We identified four decision aids and sixteen information leaflets for pregnant women. One of the decision aids [39] was excluded as it was targeted at health care professionals, not lay people. Another decision aid [40] was excluded, as it dealt with IOL, but did not explicitly cover the indications of late and post term pregnancies. Three information leaflets [41-43] were excluded, because their references to late and post-term pregnancies were confined to merely listing prolonged pregnancy as one possible indication for IOL without providing further related information.

The two decision aids and eleven information leaflets included into the assessment are listed in alphabetical order and numbered consecutively in Table 1.

**Table 1:** **Analyzed decision aids and information leaflets**

	**Provider and source of information**	**CC**	**Title**	**Year**
**Decision aids**				
1.	Midwives Information and Research Service (MIDIRS) [44] www.infochoice.org	GB	When your baby is overdue for women (W12)	2008
2.	The University of Queensland [45] www.qcmb.org.au/book/menu/information_for_parents	AU	Choosing how your labour will start in: The Having a Baby in Queensland Book, Your choices during pregnancy and birth, S. 81 - 95	2010
**Leaflets**				
3.	American Academy of Family Physicians (AAFP) [49] www.aafp.org/afp/2005/0515/p1942.html?printable = afp	US	Post-term Pregnancy: What you should know	2005
4.	American Academy of Family Physicians (AAFP) [50] http://familydoctor.org/familydoctor/en/pregnancy-newborns/labor-childbirth/pregnancy-what-to-expect-when-youre-past-your-due-date.html#	US	Pregnancy: What to expect when you’re past your due date	2000, update: 2010
5.	Bundeszentrale für gesundheitliche Aufklärung (BZgA) [46] www.familienplanung.de/schwangerschaft/die-schwangerschaft/schwangerschaftsverlauf/warten-auf-die-geburt	DE	Wenn das Baby auf sich warten lässt [When the baby keeps you waiting]	last update: 2011
6.	Institut für Qualität und Wirtschaftlichkeit im Gesundheitswesen (IQWIG) [47] http://www.gesundheitsinformation.de/wann-wird-eine-geburtseinleitung-notig.2686.de.html?part=geburt-wq-whps-xcb6	DE	Merkblatt: Überschreitung des Geburtstermins: Wann wird eine Geburtseinleitung nötig? [Beyond the due date: When is labour induction required?]	2008, update: 2012
7.	Institut für Qualität und Wirtschaftlichkeit im Gesundheitswesen (IQWIG) [48] http://www.gesundheitsinformation.de/wenn-die-geburt-des-babys-auf-sich-warten-lasst.2686.de.html?part=geburt-wq	D	Merkblatt: Wenn die Geburt des Babys auf sich warten lässt [When birth of the baby keeps you waiting]	2008, update: 2012
8.	Mayo Clinic [51] www.mayoclinic.com/health/inducing-labor/PR00117	US	Inducing labor: When to wait, when to induce	2011
9.	Mayo Clinic [52] www.mayoclinic.com/health/overdue-pregnancy/PR00116/	US	Overdue pregnancy: What to do when baby’s overdue	2011
10	National Health Service (NHS Choices) [54] http://www.nhs.uk/conditions/pregnancy-and-baby/pages/over-40-weeks-pregnant-overdue.aspx	GB	Overdue: Over 40 weeks pregnant	last review: 2011
11	National Health Service (NHS Choices) [55] http://www.nhs.uk/conditions/pregnancy-and-baby/pages/induction-labour.aspx	GB	Inducing Labor	last review: 2011
12	National Institute for Health and Clinical Excellence (NICE) [56] http://publications.nice.org.uk/ifp70	GB	Induction of labour	2008
13	UpToDate [53] http://www.uptodate.com/contents/postterm-pregnancy-beyond-the-basics?source=related_link	US	Patient information: Postterm pregnancy (Beyond the Basics)	2012

(The numeration does not indicate any ranking order).

The analysis of the decision aids and information leaflets on the basis of our checklist is displayed in Additional file 1. The reference numbers of the appraised decision aids and information leaflets quoted in brackets in the following text are consistent with the respective numeration in Table 1 and in Additional file 1.

All of the assessed decision aids and information leaflets are freely available on the respective providers’ websites except for the decision aid issued by “MIDIRS” [44] (Nr. 1) (costs: £ 11,00 in print; £ 3,60 as a downloadable PDF version).

### Decision aids

The two decision aids that we included into our assessment were from Australia, issued by the University of Queensland [45] (Nr. 2), and from the United Kingdom, issued by MIDIRS (Midwives Information and Resource Service) [44] (Nr. 1), an organization providing information resource to support the professional development of midwives. Both decision aids provide detailed information about the various options of care in cases of late and post term pregnancies. None of them gives an exact definition of a late term pregnancy, i.e. a pregnancy continuing beyond the EDD as opposed to a post term, post-date or prolonged pregnancy, i.e. a pregnancy continuing beyond 42 completed weeks. This may be owed to the fact that in Anglo-Saxon countries obstetricians – without any further implications - tend to define “term” as a period of time rather than on one particular date, as is usual in Germany.

While both decision aids explain the difficulties of the exact determination of gestational age, and describe the risks associated with late and post term pregnancies, only the British one discloses the uncertainties around the calculation of these risks.

Among the methods of stimulating labor before 42 completed weeks, the Australian decision aid only displays the membrane sweep, whereas the British one additionally describes natural (sexual intercourse, nipple stimulation) and alternative (castor oil, raspberry leaf tea, acupuncture) ways of labor stimulation.

In both publications the two basic options of “watchful waiting” and IOL are presented and the respective proceedings under each of these options are described. However, the description of the different ways of IOL in the British decision aid is comparatively short. In contrast to the Australian decision aid, the British one does not display probabilities for possible positive and negative outcomes of either option.

Whilst the British decision aid repeatedly refers to NHS guidance, the Australian decision aid refrains from any reference to guideline recommendations.

The display of the probabilities of clinical outcomes in the Australian decision aid fully complies with the IPDAS criteria. This applies in particular to the communication of risk probabilities by means of “100-person-diagrams”, which guarantee the continuity of reference parameters. For each risk probability reference is made to scientific evidence, and letters from A to C rates the quality of such evidence. In contrast, the British decision aid fully abstains from the display of risk probabilities. Contrary to the Australian publication, the British leaflet only insufficiently supports pregnant women in clarifying their personal values.

Whereas the Australian decision aid includes specific tools (structured work sheet, questionnaire) to help pregnant women clarify their preferences and discuss them with others, the British decision aid provides an empty sheet of scratch paper for this purpose. The Australian publication presents information in a balanced manner. Due to the shortness of information on pharmacological IOL as compared to alternative methods and due to the fact that the check-up methods applied under the option of “watchful waiting” and possible disadvantages of pharmacological IOL are the only text passages printed in bold, the British decision aid gives the impression of a certain bias against pharmacological IOL. Both decision aids name their authors and report their qualifications, but do not disclose whether and if so to which extent they have gone through a systematic development process including field tests with users.

Whereas the Australian decision aid comprises a detailed bibliography of scientific evidence, the British publication only refers to a corresponding publication for health care professionals [39], which then on its part contains a reference list.

Conflicts of interest are addressed in the Australian, but not in the British decision aid. Both publications are written at a generally understandable language level. The Australian decision aid distinguishes itself by its appealing layout and by clear text-supporting illustrations. The British leaflet is less clearly arranged and does not regard layout aspects to the same extent.

### Information leaflets

Of the eleven information leaflets included into the assessment, three are from Germany (Nr. 5 [46], 6 [47], 7 [48]), five from the US (Nr. 3 [49], 4 [50], 8 [51], 9 [52], 13 [53] and three from the UK (Nr. 10 [54], 11 [55], 12 [56]).

The German publications were issued by a government agency, the Bundeszentrale für gesundheitliche Aufklärung (BZgA) (Nr. 5), and by a professionally independent scientific institute under public law, the Institut für Qualität und Wirtschaftlichkeit im Gesundheitswesen (IQWIG) (Nr. 6, 7). The UK publications have been developed by the National Health Service (NHS) (Nr. 10, 11) and the National Institute of Health and Clinical Excellence (NICE) (Nr. 12), which provides national guidance and advice to improve health and social care on behalf of the NHS [52]. Of the five US publications, two have been issued by a physicians’ organization (Nr. 3, 4), two by a hospital (Nr. 8, 9) and one by a private information service for health care professionals and patients (Nr. 13).

None of the information leaflets fulfilled the IPDAS criteria for patient decision aids, which is unsurprising, as they are intended to provide mere information and are not directly targeted at involving pregnant women in the decision-making process.

Six of the eleven publications were too short and superficial to be of any substantial benefit for pregnant women, even considering the fact that they are mere information leaflets (AAFP (Nr. 3, 4, Mayo Clinic (Nr. 8, 9); NHS (Nr. 10, 11). Important features were missing in the 2–3 page publications. This concerned mainly graphs and tables on risks and probabilities, as well as detailed explanations on methods of induction. Three of the publications provided at least some important information on late and post term pregnancies (BZgA (Nr. 5), NICE (Nr. 12), UpToDate (Nr. 13) with one of them also describing CAM methods of labor stimulation (BZgA (Nr. 5). For the most part, however, the leaflets focused one-sidedly on medical and pharmacological IOL. As they were not immediately aimed at shared decision-making and did not perceive and address pregnant women in their role as decision-makers, they were not capable of facilitating a balanced choice for pregnant women based on their individual needs and values.

One of the information leaflets (IQWIG: “Überschreitung des Geburtstermins …” (“Beyond the due date …”) (Nr. 6) is confined to explaining in comprehensive language the results of a Cochrane review by Gülmezoglu et al. [11], according to which after 42 completed weeks of gestation the benefits of IOL outbalance the risks. Due to its narrow subject matter this leaflet is unsuitable for supporting pregnant women’s informed choice.

Among the mere information leaflets only the IQWIG leaflet “Wenn die Geburt des Babys auf sich warten lässt” (“When birth of the baby keeps you waiting”) (Nr. 7) stands out. The question, at what point a pregnancy is to be considered “lasting too long” is discussed in detail.

The German equivalents of late (“Terminüberschreitung”) and post term or prolonged (“Übertragung”) pregnancy are precisely defined. The methods of determining gestational age are described at length and immanent uncertainties are emphasized. “Watchful waiting” with its various check-up methods is described, however, not as one of two possible options between which pregnant women may choose, but as the initially indicated approach for healthy pregnant women beyond their EDD. Likewise, IOL is not so much presented as one option among others, but rather as the appropriate intervention to reduce perinatal mortality at least beyond 42 completed weeks of gestation. CAM methods of labor stimulation are discussed with reference to the lack or insufficiency of evidence for some of them. The pharmacological methods of IOL are described in detail. A particular paragraph is dedicated to the problem of how it may feel for a pregnant woman to be medically induced.

Outcome probabilities are presented only selectively and not comprehensively or systematically, which is why the leaflet does not allow for a balanced choice based on outcome results.

Individual values of pregnant women are not addressed. The information sheet consists of text only, the layout is little appealing and there are no illustrations. Tools to facilitate decision- making like work sheets or questionnaires are not attached. The leaflet comprises a bibliography, to which, however, no reference is made in the text, which does not allow for the attribution of the evidence provided in the bibliography to specific text passages. Only one text passage refers to a hyperlink providing the results of a study.

In particular with regard to its itemization, the leaflet is capable of supporting pregnant women in the process of decision-making, even though it is not designed as a decision aid in the proper sense. Overall, it is an item of information of good quality.

## Discussion

### Main findings

We were able to carry out a profound search and critical appraisal of patient decision aids. We could not locate a recent systematic review on evidence-based decision-aids for pregnant women on the management of term and post-term pregnancy. We did not find any (in German) or a wide variation (in English) of decision-aids on the topic that fulfilled IPDAS criteria. We were able to identify two genuine decision aids (Nr. 1, 2) referring to late and post term pregnancies. Our analysis showed that only the decision aid published by the University of Queensland (Nr. 2) complies almost entirely with the criteria of our IPDAS-based checklist. With regard to this publication only the non-disclosure of the uncertainties around the calculation of outcome risks, the lack of a reference to respective guideline recommendations and the fragmentary documentation of the development process have to be criticized. The analysis of the decision aid issued by MIDIRS (Nr. 1) has shown that the leaflet provides a great deal of helpful information, but only insufficiently complies with the IPDAS criteria, in particular with respect to the display of outcome probabilities, reference to scientific evidence within the publication itself, impartiality of information and layout aspects. The analysis of the eleven information leaflets for pregnant women has shown that only one leaflet (IQWIG, Nr. 7) is capable of facilitating pregnant women’s balanced and informed choice. As a medium of mere information, however, the leaflet – in spite of its good quality – cannot substitute a genuine decision aid, because decision aids have to include several aspects such as opportunities of value clarifications not included in common patient information.

### Strengths

The authors based their evaluation on a evidence based checklist, tailored to the decisional situation of pregnant women at and beyond term. We developed our checklist including specific informational needs of pregnant women as an important aspect of patient centered care. To our knowledge, no evidence-based high quality decision-aids (DA) for healthy pregnant women exist in German language. We consider our approach as the first DA for healthy pregnant women in our country that will be systematically developed according to international quality standards (IPDAS). This work will contribute to the systematic development of a decision-aid for healthy pregnant women on their options in term and post-term pregnancy. This procedure – regarding existing evidence and critical appraisal to look for best practice samples for the developmental process of own decision aids - might be a productive way of preparation for the development of further decision aids.

We added insights on the availability of information on CAM- options for induction of labour, combining the evaluation to a relevant preference of many patients. Our analysis showed, that CAM treatment options are rarely included in patient information or decision aids, even if they are commonly used in maternity care systems. Different authors regard the information about missing evidence as an important right of patients [17]. The new ethic standards of evidence based patient information are including the information about uncertainties and missing evidence, especially, if treatment options are widely used, like in the case of complementary and alternative medicine. So even the tools box of Patient information of the GIN-Net works define as part of patient information material the inclusion of information about the uncertainties or even missing evidence concerning treatment or screening interventions [57]. Gigerenzer and Muir Gray demand in their well known book: “We believe that the transparency reporting of both available as well as missing evidence is integral to a responsible management of uncertainty, it is an integral part of any democratic decision process, particularly when it comes to decisions about an individual health and well being!” [58] We share this opinion and therefore we included the information of CAM-options even in cases with missing evidence.

For the first time, we could combine such an evaluation for material in German language and were able to compare this to information material from other countries.

### Limitations

Related to the search, we had to limit our research to German and English languages because of feasibility, but also to the objective of this stage of our overall project which is to generate a general overview of existing relevant decisions aids in order to determine the necessity for an additional decision aid to be designed and tested for German language including CAM-options. We are aware that the evidence on many CAM options is very limited, and appreciate the difficulty to communicate this to clients. The construction of our checklist, which includes other items then used in the consent and evidence based checklist of IPDAS- criteria might be worthy of discussion. The used checklist has not been gone through a consensus process with more then four experts in gynecology, midwifery and decision aid development. However, we decided to include CAM-options as relevant patient preferences. For this we added the evidence for the needs of patients for CAM–options. For the addition of other criteria we added relevant evidence. In limiting our search to the Internet, we also considered the fact that information made available online will be capable of supporting an eminently large number of pregnant women in the process of decision-making. Hence it cannot be ruled out that further relevant decision aids may exist, which are, for instance, published in other languages or which are not available on the Internet but rather only exist in print out form at clinics or gynecologists. We could identify two good examples of decision aids in English, but not in German. We also know that decision aids are not the only way to solve decisional conflicts. In an evaluation of decision aids regarding the use of hormone replacement therapy, it was obvious that decision aids would have to be included in counseling contexts [37]. Cheyne et al. described the dilemma of pregnant women regarding the decision on whether or not to have their labours induced. They explained the difficulties, of weighting the risks of minor but frequent adverse events with IOL versus the risk of rare but very harmful adverse events (stillbirth) with expectant management [59]. They showed the need of better evidence-based patient information and better education of health care professionals in communicating the individualized risks of each treatment option. Decision aids may offer a structured procedure to support pregnant women and health care professionals to cope with uncertainties, and they should also include CAM options because many women ask for different alternatives in terms of self-activation. The actual processes of development in the discussions of the development of decision aids have to be considered during the process of decision aid development. We decided to realize an overview about already existing tools, carefully examining them to find useful samples and study the subject as deep as possible relating strength and limitations of existing tools. This way has been chosen elsewhere for the development of patient information material [60] and we used this method to prepare the development of a decision aid, but other ways to develop new decision aids have been suggested [61]. A further important step could have been put on the first place - asking the relevant target group concerning their informational needs. Meanwhile, we also developed a questionnaire and conducted a survey about informational needs of women. Results of this study will be published somewhere else and can be used for the development of a decision aid for the German target group.

For the development of a decision aid or other counseling tools, the evidence of CAM options have to be discussed and prepared in a way that pregnant women and midwives can understand and use this information.

A further limitation is the lack of evaluation studies concerning the effectiveness of identified decision aids.

### Interpretation

Clinical practice is influenced by culturally, historically, and politically aspects of its environment. This may even explain some of the differences found in national guidelines, usual routines in clinical decision-making as well as consumer involvement. It will have to be part of the reflection process of development and implementation to find out whether or not such tools may be useful within the German healthcare system. In Germany, antenatal care is free for women with a valid health insurance. Either an obstetrician or a midwife may provide it, but traditionally women attend a specialist obstetrician rather than a midwife for routine antenatal care, resulting in more than 90% of pregnant women in specialist care. The objectives of specialist-led antenatal care are risk assessment and early detection and treatment of complications [15]. Obstetricians, based on expert consensus, predominantly develop national clinical guidance in ante partum and intra partum care issued by the Association of Obstetricians and Gynecologists. In Australia and the United Kingdom, antenatal care is routinely provided by midwives and occasionally by General Practitioners. Specialist care is sought for secondary care level only. The scope of midwifery practice describes the aims of antenatal care as keeping a physiological process healthy and only then assessing pregnant women for risks. Both countries include consumer involvement in medical decision-making within their definitions of desirable antenatal and intra partum care. National (UK) or regional guidance (Australia) is developed based on a systematic process including review of evidence, multidisciplinary expert groups and consumer involvement [13,61].

## Conclusions

As an overall finding of our study it can be emphasized that the existing range of decision aids of good quality regarding care options in cases of late and post term pregnancies – at least as far as those are available on the Internet and published in English or German language - is insufficient. There is no relevant German-language decision aid available.

We therefore conclude that there is an urgent need for the development of an evidence-based decision aid of high quality regarding the options of care in cases of uncomplicated singleton pregnancies continuing beyond the EDD, particularly in German language. Also, the evidence on complementary and alternative medicine has to be translated into an understandable, evidence-based format and should then be incorporated into the development of decision aids. The common use of complementary therapies without evidence-based patient information shows the need of including CAM- options into the development of future decision support tools.

The positive example of the decision aid issued by the University of Queensland (Nr. 2) may serve as an important point of reference in the case of the development of a German decision aid including information about the existing or missing evidence concerning CAM- options. The actual evidence concerning the development process of decision aids should be taken into account. Results of this search might contribute as a first step into the framework for the design and evaluation of complex interventions presented by Craig et al. [62], which aims at ensuring the highest possible degree of evidence-basing and which divides the process of development and evaluation into several stages. The next step, the development of the decision aid, will be divided into a construction phase (definition of scope and purpose, and selection of content, framework, and format) and a pilot testing phase by interview, similar to other DA –processes [63]. A multidisciplinary steering group will supervise the process.

As we found only limited presentation of probabilities in patient information materials and decision aids, the development process will take into consideration the results of the reanalysis of development criteria regarding decision aids especially concerning the presentations of evidence [64-66].

## Additional file

Additional file 1:
**Criteria checklist and analysis of existing relevant decision aids and information leaflets.**
