# Supplementary material for: Watchful waiting or induction of labour – a matter of informed choice: identification, analysis and critical appraisal of decision aids and patient information regarding care options for women with uncomplicated singleton late and post term pregnancies: a review
Source: BMC Complement Altern Med. 2015 May 7;15:143. doi: 10.1186/s12906-015-0663-y (PMC4480447; doi:10.1186/s12906-015-0663-y)
Supplement: Additional file 1: — Criteria checklist and analysis of existing relevant decision aids and information leaflets. [file 12906_2015_663_MOESM1_ESM.docx]

**Criteria checklist and analysis of existing relevant decision aids and information leaflets**

|  | **1.** | **2.** | **3.** | **4.** | **5.** | **6.** | **7.** | **8.** | **9.** | **10.** | **11.** | **12.** | **13.** |
| --- | --- | --- | --- | --- | --- | --- | --- | --- | --- | --- | --- | --- | --- |
|  | **MIDIRS** [45] | **Univ. of Queensland** [46] | **AAFP: Postterm Pregnancy** [47] | **AAFP: Pregnancy: What to expect...** [48] | **BZgA** [49] | **IQWIG: [Beyond the due date]** [50] | **IQWIG: [When birth of the baby …]** [51] | **Mayo Clinic.: Inducing Labor** [52] | **Mayo Clinic: Overdue pregnancy** [53] | **NHS: Overdue** [54] | **NHS: Inducing Labor** [55] | **NICE** [56] | **UpToDate** [57] |
| **I. Content:** |  |  |  |  |  |  |  |  |  |  |  |  |  |
| **1.1 Does the decision aid provide information about care options in sufficient detail for decision-making?** | **✓** | **✓** | **−** | **−** | **✓−** | **−** | **✓** | **−** | **−** | **−** | **−** | **−** | **−** |
| 1.2 Does it describe the decision scenario? | **✓** | **✓** | **✓** | **✓** | **✓** | **✓** | **✓** | **✓** | **✓** | **✓** | **✓** | **✓** | **✓** |
| 1.2.1 Definition of late term pregnancy (40 + 1 to 41 + 6 weeks of gestation) | **−** | **−** | **−** | **✓** | **✓** | **✓** | **✓** | **−** | **−** | **✓** | **−** | **−** | **−** |
| 1.2.2 Definition of post term pregnancy (≥ 42 + 0 weeks of  gestation) | **✓** | **✓** | **✓** | **✓** | **✓** | **✓** | **✓** | **✓** | **✓** | **✓** | **✓** | **✓** | **✓** |
| 1.2.3 Description of the risks associated with late and post term  pregnancies | **✓** | **✓** | **✓−** | **−** | **✓−** | **✓** | **✓** | **✓** | **✓** | **✓−** | **✓−** | **−** | **✓** |
| 1.2.4 Disclosure of uncertainties associated with risk calculation | **✓** | **−** | **−** | **−** | **−** | **−** | **−** | **−** | **−** | **✓** | **✓** | **−** | **−** |

|  | **1.** | **2.** | **3.** | **4.** | **5.** | **6.** | **7.** | **8.** | **9.** | **10.** | **11.** | **12.** | **13.** |
| --- | --- | --- | --- | --- | --- | --- | --- | --- | --- | --- | --- | --- | --- |
| 1.2.5 Disclosure of risks associated with the determination of  gestational age [e. g. Naegele’s rule vs. early ultrasound scan;  focusing on the EDD (German obstetrics) vs. on a period of  delivery from 37 to 42 weeks (Anglo-Saxon obstetrics)] | **✓** | **✓** | **−** | **✓** | **−** | **−** | **✓** | **−** | **✓** | **−** | **−** | **−** | **✓** |
| 1.3. Does it specify indications of non-applicability of the decision aid (e. g. health problems of mother or fetus)? | **−** | **✓** | **−** | **−** | **−** | **✓** | **−** | **−** | **−** | **−** | **−** | **✓** | **−** |
| 1.4 Does the decision aid define its object, which is to facilitate pregnant women’s informed choice based on their personal  values? | **✓** | **✓** | **−** | **−** | **−** | **−** | **−** | **−** | **−** | **−** | **−** | **✓** | **−** |
| 1.5 Does it describe the two basic options of care? | **✓** | **✓** | **✓** | **✓−** | **✓** | **✓** | **✓** | **✓** | **−** | **✓** | **✓** | **✓−** | **✓** |
| 1.5.1 „watchful waiting“/„expectant management“ | **✓** | **✓** | **✓** | **✓** | **✓** | **✓** | **✓−** | **✓** | **−** | **✓** | **✓** | **−** | **✓** |
| 1.5.2 induction of labor (IOL) | **✓** | **✓** | **✓** | **✓** | **✓** | **✓** | **✓** | **✓** | **✓** | **✓** | **✓** | **✓** | **✓** |
| 1.5.3 Does it describe the natural birth process? | **−** | **✓** | **−** | **−** | **−** | **−** | **✓** | **−** | **−** | **−** | **−** | **−** | **✓−** |
| 1.5.4 Does it explain that most women go into spontaneous labor even beyond their EDD? | **✓** | **✓** | **−** | **−** | **✓** | **✓** | **✓** | **✓** | **✓** | **✓** | **✓** | **−** | **−** |

|  | **1.** | **2.** | **3.** | **4.** | **5.** | **6.** | **7.** | **8.** | **9.** | **10.** | **11.** | **12.** | **13.** |
| --- | --- | --- | --- | --- | --- | --- | --- | --- | --- | --- | --- | --- | --- |
| 1.6 Does it refer to natural and complementary or alternative (CAM) methods of labor stimulation? | **✓** | **✓** | **−** | **−** | **✓** | **−** | **✓** | **✓−** | **✓−** | **✓−** | **✓−** | **✓−** | **−** |
| 1.6.1 membrane sweep | **✓** | **✓** | **−** | **−** | **−** | **−** | **✓** | **−** | **−** | **✓** | **✓** | **✓** | **−** |
| 1.6.2 sexual intercourse | **✓** | **−** | **−** | **−** | **✓** | **−** | **✓** | **✓** | **✓** | **−** | **−** | **−** | **−** |
| 1.6.3 nipple stimulation | **✓** | **−** | **−** | **−** | **✓** | **−** | **✓** | **✓** | **−** | **−** | **−** | **−** | **−** |
| 1.6.4 CAM methods (e. g. castor oil, raspberry leaf tea,  acupuncture) | **✓** | **−** | **−** | **−** | **✓** | **−** | **✓** | **−** | **✓−** | **−** | **−** | **−** | **−** |
| 1.7 Does it describe the procedures of each care option in detail? | **✓** | **✓** | **−** | **−** | **✓** | **✓−** | **✓** | **−** | **✓−** | **−** | **−** | **✓−** | **✓−** |
| 1.7.1 „watchful waiting“: e. g. checking mother’s health, CTG, AFI measurement | **✓** | **✓** | **−** | **−** | **✓** | **✓** | **✓−** | **−** | **−** | **−** | **−** | **−** | **✓** |
| 1.7.2 IOL: e. g. giving of synthetic prostaglandin, giving of a  Syntocinon® infusion, breaking the waters | **✓** | **✓** | **−** | **−** | **✓−** | **−** | **✓** | **−** | **✓−** | **−** | **−** | **✓−** | **✓−** |
| 1.7.3 Does it make reference to the fact that a decision in favor of „watchful waiting“ does not foreclose a posterior decision in favor of IOL? | **−** | **✓** | **−** | **−** | **−** | **−** | **−** | **−** | **−** | **✓** | **−** | **−** | **−** |
| 1.7.4 Does it describe the positive features (benefits) of options? | **✓** | **✓** | **−** | **−** | **−** | **−** | **✓** | **−** | **−** | **−** | **−** | **−** | **−** |
| 1.7.5 Does it describe the negative features (harms, side effects,  disadvantages, risks) of options? | **✓** | **✓** | **−** | **−** | **−** | **−** | **✓** | **✓** | **−** | **✓−** | **✓−** | **−** | **−** |

|  | **1.** | **2.** | **3.** | **4.** | **5.** | **6.** | **7.** | **8.** | **9.** | **10.** | **11.** | **12.** | **13.** |
| --- | --- | --- | --- | --- | --- | --- | --- | --- | --- | --- | --- | --- | --- |
| 1.8 Does it display probabilities for positive and negative outcomes of the care options? | **−** | **✓** | **−** | **−** | **−** | **✓−** | **✓** | **−** | **−** | **−** | **−** | **−** | **−** |
| 1.9 Does it make reference to relevant guideline recommendations of medical societies? | **✓** | **−** | **−** | **−** | **−** | **−** | **✓−** | **−** | **−** | **−** | **−** | **✓** | **−** |
| **2. Does it present probabilities in an unbiased and**  **understandable way?** | **−** | **✓** | **−** | **−** | **−** | **✓−** | **−** | **−** | **−** | **−** | **−** | **−** | **−** |
| 2.1 Does it use event rates specifying the population and time  period? | **−** | **✓** | **−** | **−** | **−** | **✓** | **−** | **−** | **−** | **−** | **−** | **−** | **−** |
| 2.2 Does it compare outcome probabilities using the same  denominator, time period, scale etc.? | **−** | **✓** | **−** | **−** | **−** | **✓** | **−** | **−** | **−** | **−** | **−** | **−** | **−** |
| 2.3 Does it describe uncertainties around probabilities? | **−** | **✓** | **−** | **−** | **−** | **−** | **✓** | **−** | **−** | **−** | **−** | **−** | **−** |
| 2.4 Does it use visual diagrams? | **−** | **✓** | **−** | **−** | **−** | **−** | **−** | **−** | **−** | **−** | **−** | **−** | **−** |
| 2.5 Does it use multiple methods to view probabilities (words,  numbers, diagrams, graphs)? | **−** | **✓** | **−** | **−** | **−** | **−** | **−** | **−** | **−** | **−** | **−** | **−** | **−** |
| 2.6 Does it allow pregnant women to view probabilities based on their own situation? | **−** | **✓** | **−** | **−** | **−** | **✓** | **✓** | **−** | **−** | **−** | **−** | **−** | **−** |
| 2.7 Does it use both positive and negative frames (e. g. showing both survival and death rates)? | **−** | **✓** | **−** | **−** | **−** | **−** | **−** | **−** | **−** | **−** | **−** | **−** | **−** |

|  | **1.** | **2.** | **3.** | **4.** | **5.** | **6.** | **7.** | **8.** | **9.** | **10.** | **11.** | **12.** | **13.** |
| --- | --- | --- | --- | --- | --- | --- | --- | --- | --- | --- | --- | --- | --- |
| **3. Does the decision aid include methods for clarifying and**  **expressing pregnant women’s values?** | **✓−** | **✓** | **−** | **−** | **−** | **−** | **−** | **−** | **−** | **−** | **−** | **✓** | **−** |
| 3.1 Does it describe the procedures and outcomes to help pregnant women imagine what it is like to experience their physical,  emotional and social effects? | **✓** | **✓** | **−** | **−** | **−** | **−** | **✓** | **−** | **−** | **−** | **−** | **−** | **−** |
| 3.2 Does it ask pregnant women to consider which positive and  negative features matter most to them? | **−** | **✓** | **−** | **−** | **−** | **−** | **−** | **−** | **−** | **−** | **−** | **✓** | **−** |
| 3.3 Does it suggest ways for pregnant women to share what matters most to them with others? | **−** | **✓** | **−** | **−** | **−** | **−** | **−** | **−** | **−** | **−** | **−** | **✓** | **−** |
| **4. Does the decision aid include structured guidance in**  **deliberation and communication?** | **✓−** | **✓** | **−** | **−** | **−** | **−** | **−** | **−** | **−** | **−** | **−** | **−** | **−** |
| 4.1 Does it provide steps to make a decision? | **−** | **✓** | **−** | **−** | **−** | **−** | **−** | **−** | **−** | **−** | **−** | **✓** | **−** |
| 4.2 Does it suggest ways to talk about the decision with health care professionals? | **✓** | **✓** | **−** | **−** | **−** | **−** | **✓** | **−** | **−** | **✓** | **✓** | **✓** | **✓** |
| 4.3 Does it include tools (e. g. work sheet, questionnaire) to discuss options with others (e. g. partner, family)? | **✓−** | **✓** | **−** | **−** | **−** | **−** | **−** | **−** | **−** | **−** | **−** | **✓** | **−** |
| 4.4 Does it provide references to further sources of information? | **✓** | **✓** | **−** | **−** | **−** | **✓** | **−** | **−** | **−** | **−** | **✓** | **✓** | **✓** |
| 4.5 Does it provide references to further support services? | **−** | **✓** | **−** | **−** | **−** | **−** | **−** | **−** | **−** | **−** | **−** | **✓** | **−** |

|  | **1.** | **2.** | **3.** | **4.** | **5.** | **6.** | **7.** | **8.** | **9.** | **10.** | **11.** | **12.** | **13.** |
| --- | --- | --- | --- | --- | --- | --- | --- | --- | --- | --- | --- | --- | --- |
| **II. Development Process** |  |  |  |  |  |  |  |  |  |  |  |  |  |
| **1. Does the decision aid present information in a balanced manner?** | **✓−** | **✓** | **−** | **−** | **✓** | **−** | **✓** | **−** | **−** | **−** | **−** | **−** | **−** |
| 1.2 Does it enable pregnant women to compare the positive and negative features of options? | **✓** | **✓** | **−** | **−** | **−** | **−** | **✓** | **−** | **−** | **−** | **−** | **−** | **−** |
| 1.3 Does it show positive and negative features with equal detail? (fonts, order, display of statistics)? | **−** | **✓** | **−** | **−** | **−** | **−** | **✓** | **−** | **−** | **−** | **−** | **−** | **−** |
| **2. Has the decision aid run through a systematic development process?** | **?** | **?** | **−** | **−** | **−** | **−** | **?** | **−** | **−** | **−** | **−** | **−** | **−** |
| 2.1 Does it name its developers? | **✓** | **✓** | **−** | **−** | **−** | **−** | **−** | **−** | **−** | **−** | **−** | **−** | **✓** |
| 2.2 Does it include developers’ credentials / qualifications? | **✓** | **✓** | **−** | **−** | **−** | **−** | **−** | **−** | **−** | **−** | **−** | **−** | **✓** |
| 2.3 Has been found out what users (pregnant women and health care practitioners) need in order to discuss options? | **?** | **?** | **−** | **−** | **?** | **−** | **?** | **−** | **−** | **−** | **−** | **−** | **−** |
| 2.4 Has it been peer reviewed by pregnant women and professional experts not involved in development and field-testing? | **?** | **?** | **−** | **−** | **?** | **−** | **?** | **−** | **−** | **−** | **−** | **−** | **−** |
| 2.5 Has it been field-tested with users (pregnant women facing the decision; practitioners presenting options)? | **?** | **?** | **−** | **−** | **?** | **−** | **?** | **−** | **−** | **−** | **−** | **−** | **−** |

|  | **1.** | **2.** | **3.** | **4.** | **5.** | **6.** | **7.** | **8.** | **9.** | **10.** | **11.** | **12.** | **13.** |
| --- | --- | --- | --- | --- | --- | --- | --- | --- | --- | --- | --- | --- | --- |
| 2.6 Do the field-tests with users (pregnant women, practitioners) show that the decision aid is | **?** | **?** | **−** | **−** | **?** | **−** | **?** | **−** | **−** | **−** | **−** | **−** | **−** |
| a) acceptable, | **?** | **?** | **−** | **−** | **?** | **−** | **?** | **−** | **−** | **−** | **−** | **−** | **−** |
| b) balanced for undecided pregnant women, | **?** | **?** | **−** | **−** | **?** | **−** | **?** | **−** | **−** | **−** | **−** | **−** | **−** |
| c) understood by those with limited reading skills as well? | **?** | **?** | **−** | **−** | **?** | **−** | **?** | **−** | **−** | **−** | **−** | **−** | **−** |
| **3. Does the decision aid use up to date scientific evidence that is cited in a reference section or bibliography?** | **−** | **✓** | **−** | **−** | **−** | **✓−** | **✓−** | **−** | **−** | **−** | **−** | **−** | **✓−** |
| 3.1 Does it provide references to evidence used? | **−** | **✓** | **−** | **−** | **−** | **✓** | **✓** | **−** | **−** | **−** | **−** | **−** | **✓** |
| 3.2 Does it describe the quality of scientific evidence (including lack of evidence)? | **−** | **✓** | **−** | **−** | **−** | **−** | **−** | **−** | **−** | **−** | **−** | **−** | **−** |
| 3.3 Does it use evidence from studies with pregnant women similar to those of the target audience? | **−** | **✓** | **−** | **−** | **−** | **✓** | **✓** | **−** | **−** | **−** | **−** | **−** | **−** |
| 3.4 Does it report the date of its last update? | **✓** | **✓** | **−** | **✓** | **−** | **✓** | **✓** | **−** | **−** | **✓** | **✓** | **−** | **✓** |
| 3.5 Does it report how often the decision aid is updated? | **✓** | **✓** | **−** | **−** | **−** | **✓** | **✓** | **−** | **−** | **✓** | **✓** | **−** | **−** |

|  | **1.** | **2.** | **3.** | **4.** | **5.** | **6.** | **7.** | **8.** | **9.** | **10.** | **11.** | **12.** | **13.** |
| --- | --- | --- | --- | --- | --- | --- | --- | --- | --- | --- | --- | --- | --- |
| **4. Does the decision aid disclose conflicts of interest?** | **−** | **✓** | **−** | **−** | **−** | **−** | **−** | **−** | **−** | **−** | **−** | **−** | **✓−** |
| 4.1 Does it report any source of funding to develop and distribute the patient decision aid? | **−** | **✓** | **−** | **−** | **−** | **−** | **−** | **−** | **−** | **−** | **−** | **−** | **✓** |
| 4.2 Does it report whether authors or their affiliations stand to gain or lose by choices pregnant women make after using the patient decision aid? | **−** | **✓** | **−** | **−** | **−** | **−** | **−** | **−** | **−** | **−** | **−** | **−** | **−** |
| **5. Does the decision aid use plain language?** | **✓** | **✓** | **✓** | **✓** | **✓** | **✓** | **✓** | **✓** | **✓** | **✓** | **✓** | **✓** | **✓** |
| 5.1 Is it written at a level that can be understood by the majority of pregnant women? | **✓** | **✓** | **✓** | **✓** | **✓** | **✓** | **✓** | **✓** | **✓** | **✓** | **✓** | **✓** | **✓** |
| 5.2 Does it provide ways to help pregnant women understand information other than reading (audio, video, in-person discussion)? | **−** | **−** | **−** | **−** | **−** | **−** | **−** | **−** | **−** | **✓** | **✓** | **✓** | **−** |
| 5.3 Does it consider cultural particularities (e. g. foreign language versions for pregnant women with migration background)? | **−** | **−** | **−** | **−** | **−** | **−** | **−** | **−** | **−** | **−** | **−** | **−** | **−** |
| 5.4 Does it consider layout aspects (e. g. font design, line format, overall design)? | **✓−** | **✓** | **−** | **−** | **−** | **−** | **−** | **−** | **−** | **−** | **−** | **−** | **−** |
| 5.5 Is it stringently composed and clearly structured and arranged? | **✓** | **✓** | **✓** | **✓** | **✓** | **✓** | **✓** | **✓** | **✓** | **✓** | **✓** | **✓** | **✓** |

**Legend:**

| **✓** | Criterion fulfilled |
| --- | --- |
| **✓−** | Criterion partially fulfilled |
| **−** | Criterion not fulfilled |
| **?** | Criterion not checkable |
